# Concurrent of compound heterozygous variant of a novel in-frame deletion and the common hypomorphic haplotype in *TBX6* and inherited 17q12 microdeletion in a fetus

**DOI:** 10.1186/s12884-024-06653-2

**Published:** 2024-07-01

**Authors:** Guoqiang Li, Yiyao Chen, Xu Han, Niu Li, Shuyuan Li

**Affiliations:** 1 Department of Reproductive Genetics, International Peace Maternity and Child Health Hospital, School of Medicine, Shanghai Jiao Tong University, No.910, Hengshan Road, Shanghai, 200030 China; 2 Shanghai Key Laboratory of Embryo Original Diseases, Shanghai, 200030 China; 3 Faculty of Medical Laboratory Science, College of Health Science and Technology, School of Medicine, Shanghai Jiao Tong University, Shanghai, 200025 China

**Keywords:** Congenital scoliosis, TBX6, Novel variant, Functional study, 17q12 microdeletion

## Abstract

**Background:**

*TBX6*, a member of the T-box gene family, encodes the transcription factor box 6 that is critical for somite segmentation in vertebrates. It is known that the compound heterozygosity of disruptive variants *in trans* with a common hypomorphic risk haplotype (T-C-A) in the *TBX6* gene contribute to 10% of congenital scoliosis (CS) cases. The deletion of chromosome 17q12 is a rare cytogenetic abnormality, which often leads to renal cysts and diabetes mellitus. However, the affected individuals often exhibit clinical heterogeneity and incomplete penetrance.

**Methods:**

We here present a Chinese fetus who was shown to have CS by ultrasound examination at 17 weeks of gestation. Trio whole-exome sequencing (WES) was performed to investigate the underlying genetic defects of the fetus. In vitro functional experiments, including western-blotting and luciferase transactivation assay, were performed to determine the pathogenicity of the novel variant of *TBX6*.

**Results:**

WES revealed the fetus harbored a compound heterozygous variant of c.338_340del (p.Ile113del) and the common hypomorphic risk haplotype of the *TBX6* gene. In vitro functional study showed the p.Ile113del variant had no impact on TBX6 expression, but almost led to complete loss of its transcriptional activity. In addition, we identified a 1.85 Mb deletion on 17q12 region in the fetus and the mother. Though there is currently no clinical phenotype associated with this copy number variation in the fetus, it can explain multiple renal cysts in the pregnant woman.

**Conclusions:**

This study is the first to report a Chinese fetus with a single amino acid deletion variant and a T-C-A haplotype of *TBX6*. The clinical heterogeneity of 17q12 microdeletion poses significant challenges for prenatal genetic counseling. Our results once again suggest the complexity of prenatal genetic diagnosis.

**Supplementary Information:**

The online version contains supplementary material available at 10.1186/s12884-024-06653-2.

## Introduction

Congenital scoliosis (CS) is characterized by the lateral curvature of the spine resulting from vertebral anomalies that arise during intrauterine somitogenesis [1]. The estimated incidence of CS is 0.5–1/1,000 live births [2]. Highly synchronized developmental molecular pathways are involved in somitogenesis, with the Notch signal transduction pathway being crucial during early embryogenesis. The pathogenic variants of genes (*MESP2*, *DLL3*, *LFNG*, and *HES7*) in the Notch signaling pathway have been implicated in spondylocostal dysostosis (SCD) [3].

The transcription factor box 6 (*TBX6*), a member of the phylogenetically conserved T-box gene family, encodes transcription factors known for their highly conserved N-terminal DNA-binding domain (T-box), which plays a critical role in somite segmentation in vertebrates. Approximately 10% of CS cases can be attributed to the compound heterozygosity of null mutations (such as a deletion copy number variation (CNV) or a loss-of-function variant allele) *in trans* with a common hypomorphic risk haplotype (T-C-A) [4–6]. This haplotype is delineated by single nucleotide polymorphisms (SNPs) at the positions of rs2289292, rs3809624, and rs38090627 within the *TBX6* gene. Liu et al. defined the specific clinical disease entity (TBX6-Associated CS, TACS) which was associated with a combination of a loss-of-function *TBX6* lesion and a T-C-A haplotype *in trans*, as an underlying genetic etiology [7]. In addition, the phenotypes observed from the combined null and hypomorphic alleles of *Tbx6* mouse model are similar to those in human CS [8]. Subsequent studies have elucidated the mechanism of TACS, by showing the direct interaction of Tbx6 with the *Mesp2* upstream region to mediate Notch signaling in the anterior presomitic mesoderm during somitogenesis [9].

Deletions of chromosome 17q12, including that of the *HNF1B* gene, result in 17q12 microdeletion syndrome (OMIM#614,527), which have been associated with kidney abnormalities, maturity-onset diabetes of the young type 5 (MODY5), and neurodevelopmental or neuropsychiatric disorders. Though the CNV in most patients occurs *de novo*, about 25% of patients are inherited from their parents [10].

Herein, we report a compound heterozygous variant of a novel in-frame deletion and the common hypomorphic haplotype in *TBX6* in a Chinese fetus with CS. Meanwhile, the fetus was found having a pathogenic CNV involving 17q12 region inherited from the mother.

## Patient and methods

### Patient

A 33-year-old primigravid woman was referred to Department of Prenatal Diagnosis for evaluation and counseling of fetal spine abnormalities. The pregnant woman and her 32-year-old husband are non-consanguineous Han Chinese, and neither has a family history of spinal related skeletal abnormalities. Amniocentesis was conducted at 19 weeks of gestation for the patient and the samples were analyzed by conventional karyotyping, chromosomal microarray analysis (CMA), and trio whole-exome sequencing (trio-WES). This study was approved by the Ethics Committee of the International Peace Maternity and Child Health Hospital (2022-14), and written informed consents were obtained from the family.

### Whole-exome sequencing and Sanger sequencing

The karyotyping and CMA of fetal samples were conducted as described previously [11]. For trio-WES, genomic DNA was extracted from amniotic fluid and peripheral blood samples of the parents using the MagPure Buffy Coat DNA Midi KF Kit (MAGEN) according to the manufacturer’s instructions. 300 ng of DNA underwent shearing to produce 100–500 bp fragments. Fragments of 200–300 bp were selectively retained using VAHTS DNA clean beads (Vazyme, Nanjing, Jiangsu, China). The sample library was subjected to hybridization with customized gene fragment capture probes (KAPA HyperExome, Roche) according to the manufacturer’s instructions. Subsequently, the captured library was sequenced on the MGISEQ-2000 platform (MGI, Shenzhen, Guangdong, China) in accordance with the manufacturer’s instructions.

The sequencing quality of the raw data was evaluated using the SOAPnuke software, with the removal of low-quality and contaminated reads to obtain clean reads. The clean reads were aligned to the GRCh37/hg19 human reference sequence using the Burrows-Wheeler Aligner. Single nucleotide variants, insertions, and deletions were identified using the GATK software package. The annotation and screening of variants were performed using the BGI-Varanno algorithm. The ExomeDepth software was used for exon CNV detection, while the CNV kit software was used for the CNV detection of large segments exceeding 1 M.

Additionally, exon 3 of *TBX6* and rs2289292, rs3809624, and rs3809627 were amplified via polymerase chain reaction from genomic DNA (primer sequences available upon request). Sanger sequencing was performed on an ABI 3700 sequencer (Applied Biosystems, Foster City, CA, USA).

### In silico analysis of the TBX6‑Ile113del variant

Conservation analysis of TBX6‑Ile113 was performed using Sequence Manipulation Suite: Color Align Properties (http://www.bioinformatics.org/sms2/color_align_prop.html). Since TBX6 crystal structure was not available, the three-dimensional (3D) structure of the wild-type (WT) TBX6 protein was simulated using Pymol v.1.8.4.0 software (https://www.pymol. org) using the amino acid sequence of TBX3 (NP_005987.3).

### Plasmids construction

Wild-type (WT) and p.Ile113del mutant *TBX6* cDNAs were synthesized by BioVision (Shanghai, China) and cloned into the pcDNA3.1-Flag vector using the Hieff Clone Plus One Step Cloning Kit (Yeasen Biotechnology Co., Ltd., Shanghai, China), following the manufacturer’s instructions. The TBX6-p.Met111Ile mutant constructs was generated using a Mut Express II Fast Mutagenesis Kit V2 (Vazyme, Nanjing, Jiangsu, China). A 300-bp *MESP2* promoter sequence was synthesized by BioVision and cloned into the pGL3-basic vector (Promega, Madison, WI, USA) to generate a luciferase reporter plasmid (MESP2-P2L-300-luc). Verification of all vectors was carried out through Sanger sequencing.

### Cell culture and transient transfection

Human embryonic kidney (HEK) 293T cells were obtained from the ATCC (American Type Culture Collection, Manassas, VA, USA). 293T cells were cultured in Dulbecco’s modified Eagle’s medium (Corning, NY, USA), supplemented with 10% (v/v) fetal bovine serum (BioVision Technology (Shanghai) Co., Ltd., Shanghai, China) and 1% penicillin/streptomycin (Beyotime Biotechnology, Shanghai, China) in a 5% CO_2_ incubator at 37 °C. Transfection was performed using jetPRIME transfection reagent (Polyplus Transfection, Illkirch, France).

### Western blot analysis

HEK 293T cells were seeded (5 × 10^5^ cells/well) in 12-well plates and transfected with 0.8 µg of the designated TBX6 constructs. At 36 h post-transfection, proteins were extracted from whole-cell lysates, separated on 10% sodium dodecyl-sulfate polyacrylamide gel electrophoresis gels, transferred to polyvinylidene difluoride membranes, and incubated with rabbit anti-Flag polyclonal antibody (Proteintech, Wuhan, Hubei, China). Protein detection was achieved using a chemiluminescence system with a horseradish peroxidase-conjugated secondary antibody. β-actin was used as a control for ensuring consistent loading.

### Luciferase assay

The MESP2-P2L-300-luc plasmid was employed to assess the regulatory role of TBX6 in transcriptional modulation. Briefly, HEK293T cells were seeded in 96-well plates at a density of 4 × 10^4^ per well. After 24 h of culture, the cells were co-transfected with 80 ng of MESP2-P2L-300-luc, 1 ng of pRL-SV40, and 20 ng of control or TBX6 expression vectors. Subsequently, 24 h after transfection, the luciferase activity was measured using the Dua-Glo luciferase assay system (Promega Corporation, Madison, WI, USA) and normalized to Renilla luciferase encoded by pRL-SV40.

## Results

### Description of the fetus

**Fig. 1:**
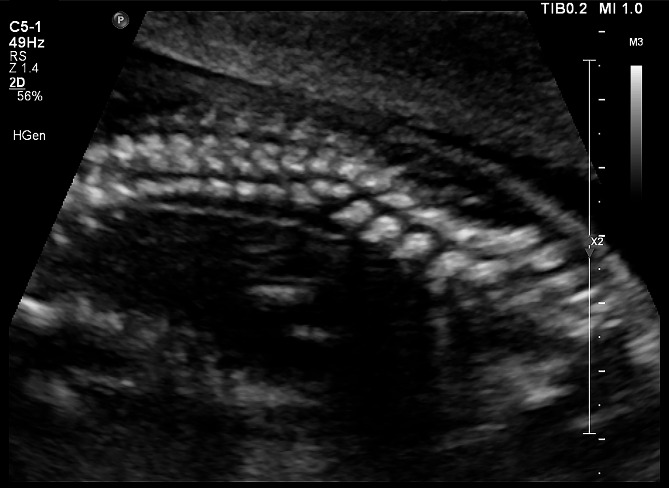
Ultrasound scan of the fetus at 17 weeks of gestation

The fetus was the first pregnancy of the family, and there was no special in the prenatal examination before 12 weeks of gestation. At 13 weeks of gestation, the fetus was first shown to have abnormal spinal development by ultrasound examination at local hospital. A detailed fetal ultrasound scan at 17 weeks of gestation at our hospital revealed abnormal development of the spinal cord, unusual arrangement of the local vertebrae in the thoracic and sacrococcygeal segments, and scoliosis **(**Fig. 1**)**. No abnormalities were found in other organs of the fetus. After receiving detailed genetic counseling about the results of trio-WES and CMA, the pregnant woman chose to terminate the pregnancy at 23 weeks. The autopsy was conducted and the results confirmed that the fetus had scoliosis (to the left), involving the thoracic_8 to lumbar_5 segments, accompanied by lumbar_5 vertebral hypertrophy.

### Identification and in silico analysis of the *TBX6* variants in the patient

**Fig. 2:**
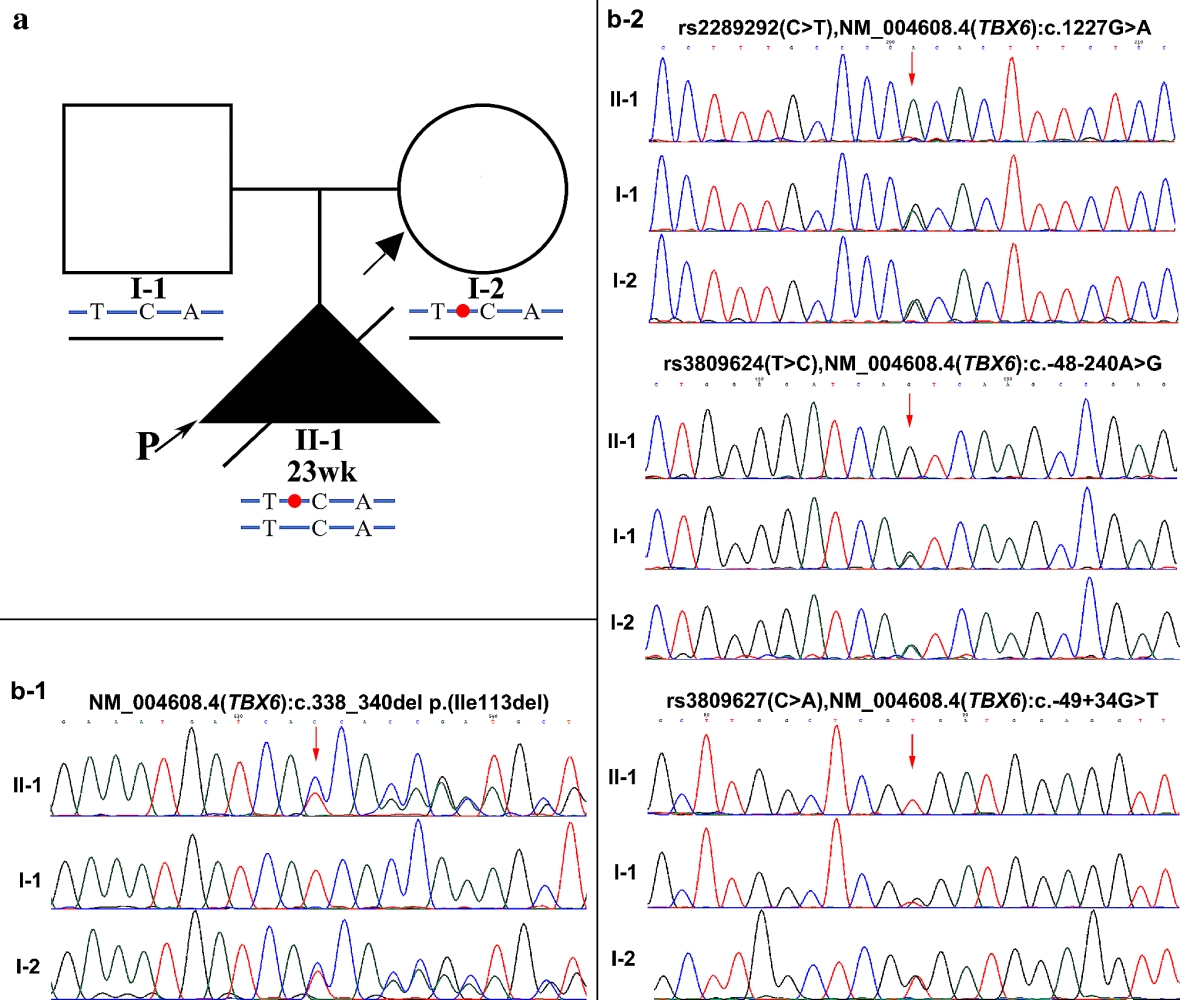
Pedigree and chromatogram of Sanger sequencing analysis of a Chinese family. (**a**) Family pedigree. (**b**-1) A heterozygous *TBX6* variant (c.338_340del, p.Ile113del) in the fetus and the mother. (**b**-2) The homozygous common hypomorphic allele in the fetus, and the heterozygous common hypomorphic allele in the mother and father. Red arrows indicate the corresponding sequences where the mutation was found

To explore the underlying genetic variants of the fetus, trio-WES was performed in the family. After excluding low-confidence variants, high-frequency variants with an allele frequency above 1% in the gnomAD database, and benign variants predicted by REVEL [12], a heterozygous in-frame deletion variant [NM_004608.4: c.338_340del, p.(Ile113del)] and a well-known homozygous hypomorphic allele (T-C-A) in *TBX6* gene were identified in the fetal genome. Sanger sequencing revealed that the c.338_340del variant was inherited from the mother, and both parents are heterozygous carriers of the hypomorphic allele (Fig. 2). Analysis of the sequencing data showed that the two heterozygous variants are *cis* in the mother.

**Fig. 3:**
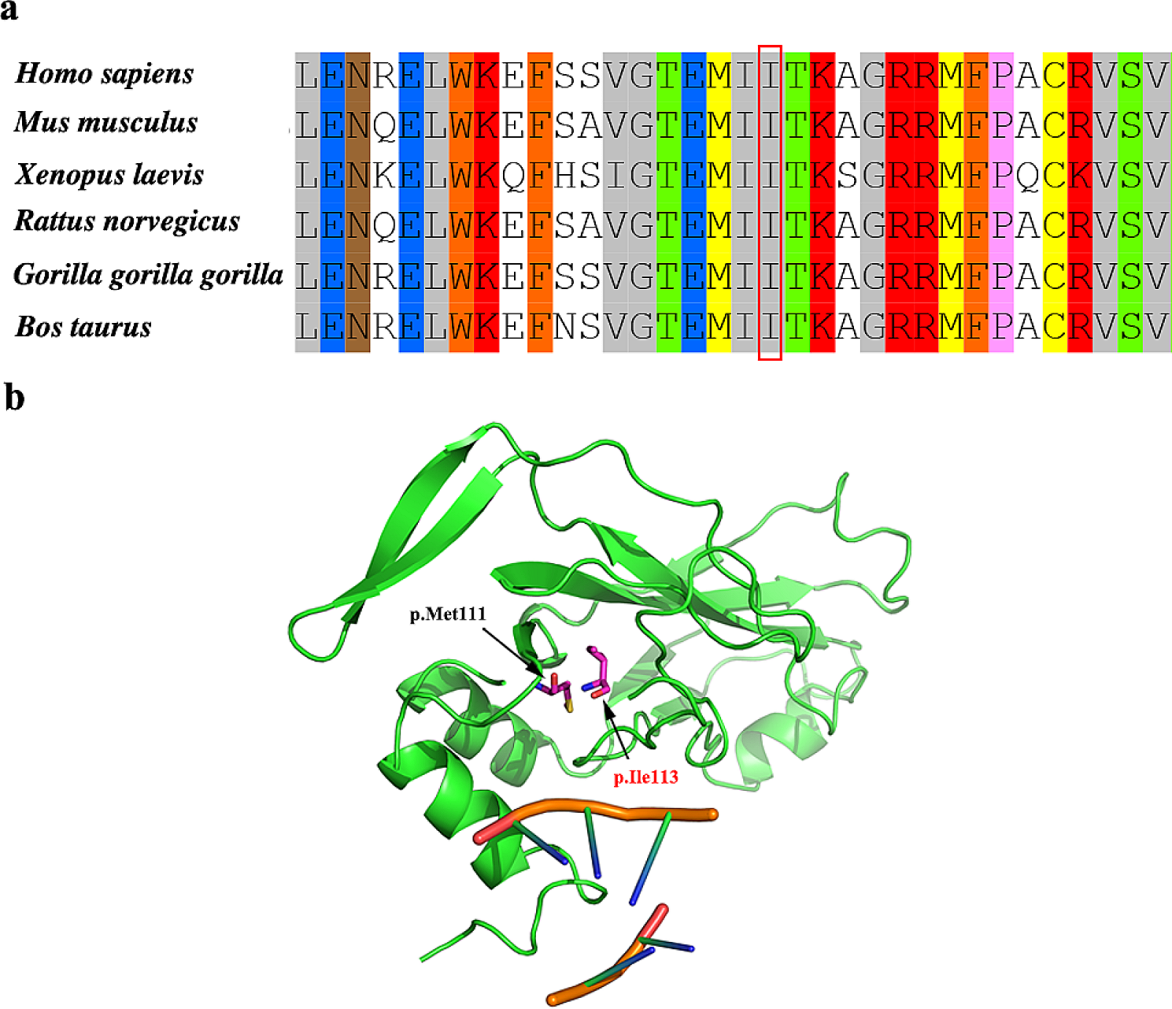
(**a**) Conservation analysis of TBX6. Alignment of amino acid sequences of TBX6 in different species. The circled residues represent the corresponding residues of TBX6 p.Ile113. (**b**)Space distribution of the two variants evaluated in this study. A T-box domain model of TBX6 (89–273 amino acids) in complex with DNA was built using the Swiss-Model with the crystal structure of TBX3 (PDB ID code: 1H6F) as a reference. The the two variants (p.Met111Ile and p.Ile113del) reside in the T-box domain of the TBX6 protein, and are in close proximity to the target DNA

The TBX6 p.Ile113del variant has not been reported previously and was not included in the known public databases (i.e., gnomAD, HGMD, and ClinVar). Ile113 resides within the T-box domain (DNA-binding region) of TBX6 and is highly conserved in multiple species (Fig. 3a). Predictive assessments using various in silico tools, including MutationTaster2 and MutPred-Indel, indicated the deleterious nature of the p.Ile113del variant. In addition, the crystal structure analysis using TBX3 homologous sequence showed that the p.Ile113del variant likely impaired its DNA-binding ability (Fig. 3b).

### Impairment of the transcriptional activity of TBX6 by the Ile113del variant

**Fig. 4:**
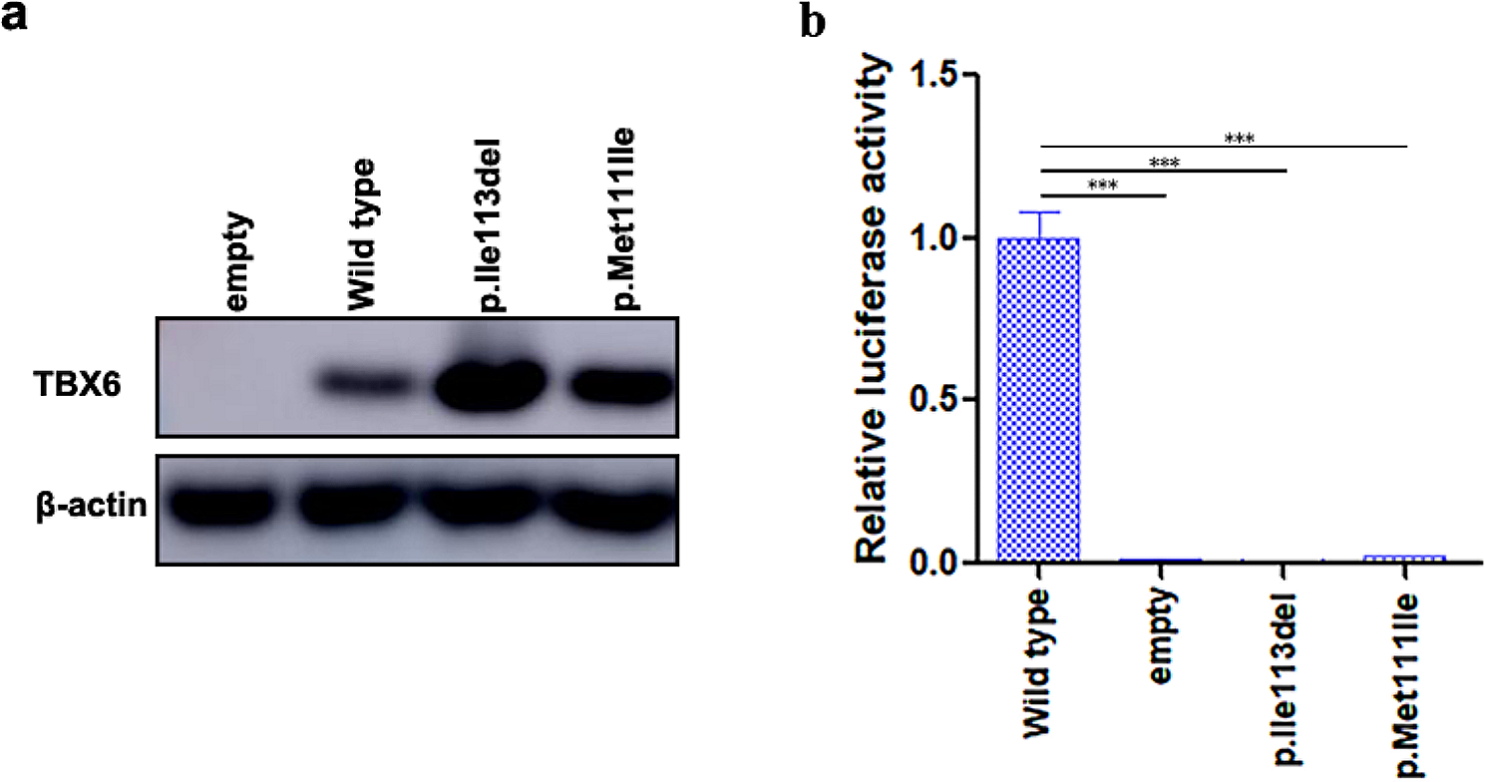
Results of the western blot and luciferase assay. (**a**) The expression of the WT and mutant Flag-TBX6 was evaluated using a western blot assay. (**b**) Luciferase transactivation assays of the WT and mutant Flag-TBX6 using MESP2 promoter reporter. Triple asterisks (***) indicate p values less than 0.0001

To assess the impact of the p.Ile113del variant on TBX6 function, a luciferase assay was performed using a reporter construct containing a fragment of the *MESP2* promoter as described [3]. In addition to the TBX6 p.Ile113del variant, a previously reported deleterious missense variant (Met111Ile), located close to Ile113, was also constructed as a positive control [6]. In vitro experiments showed that these two variants did not impair the protein expression of TBX6, but rather upregulated the protein expression levels to varying degrees (Fig. 4a). As expected, the Met111Ile mutant displayed extremely low transcriptional activity, suggesting that the functional evaluation system was appropriate. Compared with WT-TBX6, the Ile113del variant almost resulted in complete loss of its transcriptional activity (Fig. 4b). Together, these results indicate that although the Ile113del mutant protein only loses one amino acid, its impact on TBX6 is similar to those of null variants (i.e., nonsense variants), greatly impairing its transcriptional activity. According to the guidelines recommended by the American College of Medical Genetics and Genomics (ACMG) for the interpretation of sequence variants [13], the variant (c.338_340del) was classified as likely pathogenic (PS3_Supporting + PM1 + PM2_Supporting + PM4 + PP4). This in-frame deletion variant, together with a known hypomorphic haplotype (T-C-A) in *TBX6* could explain the CS in the fetus.

### Identification of 17q12 microdeletion in the family as a secondary finding

**Fig. 5:**
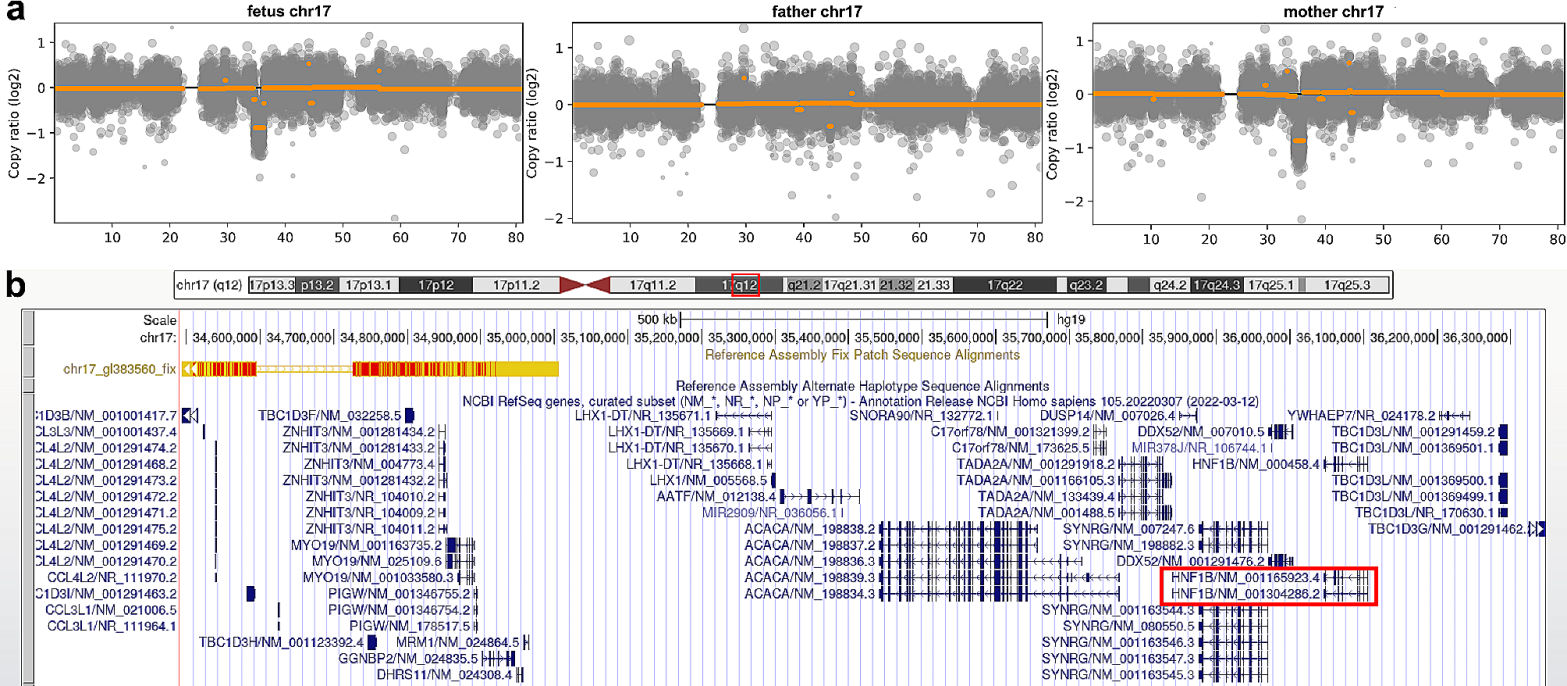
Copy number variation analysis in this family. (**a**) Sequencing depth analysis using the WES data showed a 17q12 deletion (hg19, chr17:34493374–36,346,847) in the fetus and mother harbored. (**b**) Genes involved in the 17q12 region

It has been reported that approximately 5% of the patients have more than one molecular finding [14]. We conducted routine analysis of CNVs for each sample by comparing the read depth with WES data from other samples of the same batch. By this strategy, we identified a 1.85 Mb deletion on 17q12 region (hg19, chr17:34493374–36,346,847) in the fetus and the mother (Fig. 5a), which was also identified by CMA. This CNV can lead to 17q12 microdeletion syndrome, mainly manifested as renal cysts and diabetes syndrome caused by haploinsufficiency of *HNF1B* gene (chr17:34815072–36,192,489, OMIM#189,907) (Fig. 5b) [15]. According to the clinical interpretation guidelines of the American College of Medical Genetics and Genomics and the Clinical Genome [16], the microdeletion of 17q12 was classified as pathogenic.

Since the ultrasound examination did not indicate any renal abnormalities in the fetus, we therefore evaluated the health status of the mother. Ultrasound reports from another hospital showed the mother had multiple cystic areas in both kidneys with multiple stones, suggesting cystic changes in the renal medulla, the possibility of a sponge kidney, and several anechoic areas in the right kidney, indicative of multiple renal cysts.

## Discussion

In this study, we reported a Chinese fetal patient with spinal abnormalities who was concurrent of compound heterozygous variants of a novel in-frame deletion (c.338_340del, p.Ile113del) and the common hypomorphic haplotype in *TBX6* and a 1.85 Mb deletion on 17q12 region (hg19, chr17:34493374–36,346,847). Postnatal patients with TACS have been widely reported in Han Chinese, Japanese and European cohorts, and a clinical diagnostic algorithm (TACScore) had been developed [7]. The affected individuals can present with a range of spinal deformity from simple hemivertebrae/butterfly vertebrae involving the lower part of the spine (T8-S5), with or without simple rib anomaly to a more severe clinical phenotype, defined as spondylocostal dysostosis (SCD) that involves multiple segmentation defects of the vertebrae with malalignment of the ribs [7]. In contrast, few prenatal cases were reported. In 2020, Errichiello et al. reported a SCD fetus with severe kyphoscoliosis involving the entire spine and segmentation abnormalities (hemivertebrae) accompanied by asymmetric rib defects, who harbored a homozygous *TBX6* stop-gained variant [NM_004608.3:c.1148 C > A, p.(Ser383Ter)] [17]. And in 2024, Liu et al. reported five fetuses with vertebral malformations (such as hemivertebra, butterfly vertebra, and scoliosis), who were detected with 16p11.2 deletion and the *TBX6* haplotype of T-C-A. However, no detailed phenotypic description was available and the clinical information was limited [18]. The understanding of the prenatal phenotype of TACS is still limited and requires more collection of fetal patients.

In reported studies of TACS, the rare null mutations of *TBX6* include 16p11.2 deletion, frameshift variants, nonsense variants, splicing variants, and missense variants. To the best of our knowledge, no deleterious single amino acid deletion variant has been reported in patients with TACS. The present study reported the first pathogenic single amino acid deletion variant of *TBX6* (c.338_340del, p.Ile113del). Assessing the pathogenicity of single amino acid deletion variants, akin to missense variants, poses challenges not encountered with apparent null variants. Therefore, conducting functional research is important for evaluating the variants. Given its role as a transcription factor, the transcriptional assay was suitable for the assessment of functional change in TBX6. We here clearly showed the Ile113del variant led to the loss of TBX6 transcriptional activity, indicating its characterization as a null variant. Additionally, analysis of the 3D structure of TBX6 showed that the Ile113 and Met111 are very close and both have the ability to directly bind to DNA (Fig. 3), which implies that the functional changes of Ile113del may be in line with that of the deleterious variant in the 111 residue. The results from luciferase report assays have well confirmed this hypothesis (Fig. 4). Considering the time challenge of functional experiments in prenatal diagnosis, we emphasize deepening our understanding of variant pathogenicity through structural analysis.

TACS is caused by bi-allelic variants of *TBX6*, consistent with autosomal recessive trait manifestation. The only exception was an autosomal dominant SCD caused by *TBX6* elongation (c.1311 A > T, p.*437Cext*81) variant reported in a Macedonian family, whose pathogenic mechanism remained to be explored. To explain the heterogeneity of the TACS clinical phenotype, an allelic heterogeneity hypothesis of *TBX6* was proposed. For the heterozygous common risk haplotype of *TBX6*, it causes only a moderate reduction in *TBX6* expression. At the same time, the *TBX6* mutations in the other allele lead to different dosage effects, thus causing variation of phenotypic severity [19].

In addition to the compound heterozygosity in *TBX6*, the fetus harbored a microdeletion of 17q12 inherited from the mother. The 17q12 deletion syndrome can lead to a variety of clinical phenotypes after birth, mainly including congenital abnormalities of the kidney and urinary tract (genital tract abnormalities, renal cysts, abnormalities of kidney parenchyma, fusion anomalies), MODY5, and neurodevelopmental or neuropsychiatric disorders (developmental delay, intellectual disability, autism spectrum disorder, schizophrenia) [10]. However, the knowledge about the prenatal phenotypes of 17q12 deletion syndrome is still limited. The results of ultrasound screening at gestational weeks of 20–32 showed that the most common prenatal phenotypes were kidney anomalies, including renal hyperechogenicity and multicystic dysplastic kidneys [20, 21]. Although ultrasound scan of the fetus in this study at 17 weeks of gestation found no abnormalities on kidneys, it was possible to appear kidney anomalies after 20 weeks. Moreover, it was reported that the penetrance of 17q12 deletion was 34.4% (13.7-70.0%) [22], which posed challenges for prenatal genetic counseling. The 17q12 deletion syndrome is not involved in congenital scoliosis. Therefore, the CNV of the 17q12 deletion was identified as the cause of the mother’s manifestation of multiple renal cysts, with no discernable connection with the congenital scoliosis of the fetus.

In conclusion, our study identified a novel *TBX6* variant with a common risk haplotype and elucidated the damaging effect of the indel variant on the TBX6 protein via in vitro experiments. This study is the first to report a Chinese patient with TACS during the fetal period, which broadens the mutation spectrum of the *TBX6* gene and extends the genotype-phenotype relationship in the study of TACS. In addition, we yet reported a pathogenic CNV with clinical penetrance in the fetus, indicating once again the complexity of prenatal diagnosis and genetic counseling.

### Electronic supplementary material

Below is the link to the electronic supplementary material.

Supplementary Material 1

## Data Availability

The raw data are available from the corresponding author on reasonable request.

## Abbreviations

TBX6: Transcription Factor Box 6
CS: Congenital Scoliosis
SCD: Spondylocostal Dysostosis
TACS: TBX6-Associated Congenital Scoliosis
CMA: Microarray Analysis
WES: Whole-Exome Sequencing
CNV: Copy Number Variation
SNPs: Single Nucleotide Polymorphisms
3D: Three-Dimensional
WT: Wild-Type
HEK: Human Embryonic Kidney
MODY5: Maturity-Onset Diabetes Of The Young Type 5
