# Supplementary figures and images for: Concurrent of compound heterozygous variant of a novel in-frame deletion and the common hypomorphic haplotype in TBX6 and inherited 17q12 microdeletion in a fetus
Source: BMC Pregnancy Childbirth. 2024 Jul 1;24:456. doi: 10.1186/s12884-024-06653-2 (PMC11218386; doi:10.1186/s12884-024-06653-2)

Supplementary Information: full-length blots of **Fig. 4a**

TBX6:

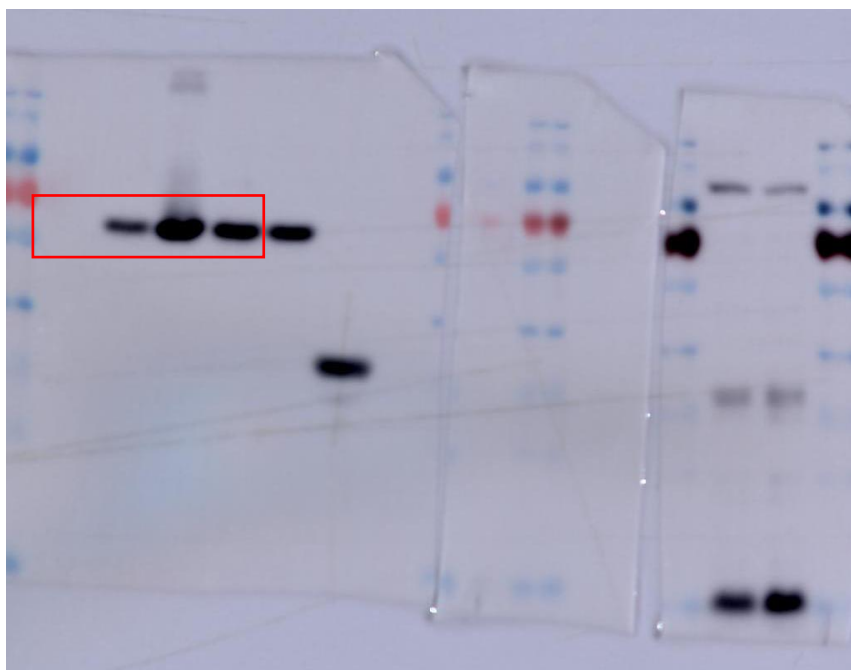

$\beta$ -actin:

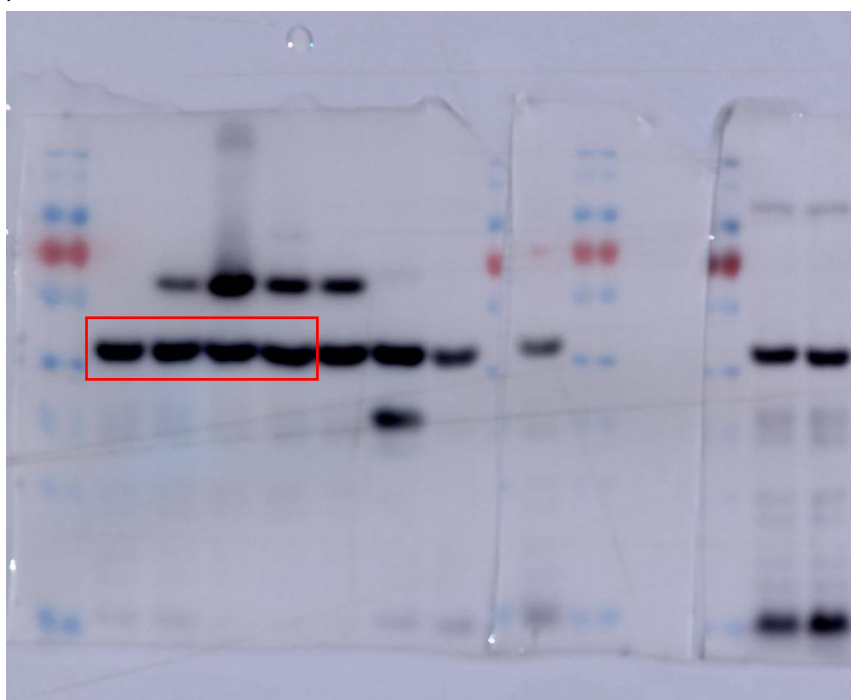

Supplement: Supplementary file 1 — Supplementary Material 1 [file 12884_2024_6653_MOESM1_ESM.pdf]
